# Vitamin D and Ceramide Metabolomic Profile in Acute Myocardial Infarction

**DOI:** 10.3390/metabo14040233

**Published:** 2024-04-18

**Authors:** Melania Gaggini, Federica Marchi, Nataliya Pylypiv, Alessandra Parlanti, Simona Storti, Umberto Paradossi, Sergio Berti, Cristina Vassalle

**Affiliations:** 1 Institute of Clinical Physiology, National Research Council, Via G. Moruzzi 1, 56124 Pisa, Italy; melania.gaggini@cnr.it; 2 Fondazione CNR-Regione Toscana Gabriele Monasterio, Ospedale G Pasquinucci, 54100 Massa, Italy; federica.marchi@ftgm.it (F.M.); pylypivn@ftgm.it (N.P.); alessandra.parlanti@ftgm.it (A.P.); simona.storti@ftgm.it (S.S.); umberto.paradossi@ftgm.it (U.P.); berti@ftgm.it (S.B.); 3 Fondazione CNR-Regione Toscana Gabriele Monasterio, Via G. Moruzzi 1, 56124 Pisa, Italy

**Keywords:** vitamin D, 25(OH)D, sphingolipids, ceramides, acute myocardial infarction, cardiovascular risk

## Abstract

Sphingolipids (SLs) influence several cellular pathways, while vitamin D exerts many extraskeletal effects in addition to its traditional biological functions, including the modulation of calcium homeostasis and bone health. Moreover, Vitamin D and SLs affect the regulation of each others’ metabolism; hence, this study aims to evaluate the relationship between the levels of 25(OH)D and ceramides in acute myocardial infarction (AMI). In particular, the blood abundance of eight ceramides and 25(OH)D was evaluated in 134 AMI patients (aged 68.4 ± 12.0 years, 72% males). A significant inverse correlation between 25(OH)D and both Cer(d18:1/16:0) and Cer(d18:1/18:0) was found; indeed, patients with severe hypovitaminosis D (<10 ng/mL) showed the highest levels of the two investigated ceramides. Moreover, diabetic/dyslipidemic patients with suboptimal levels of 25(OH)D (<30 ng/mL) had higher levels of both the ceramides when compared with the rest of the population. On the other hand, 25(OH)D remained an independent determinant for Cer(d18:1/16:0) (STD Coeff −0.18, *t*-Value −2, *p* ≤ 0.05) and Cer(d18:1/18:0) (−0.2, −2.2, *p* < 0.05). In light of these findings, the crosstalk between sphingolipids and vitamin D may unravel additional mechanisms by which these molecules can influence CV risk in AMI.

## 1. Introduction

Vitamin D, a fat-soluble vitamin with a steroidal nature (secosteroid), is mainly obtained through skin exposure to UV irradiation (with only a small percentage derived from food intake), where the conversion of 7-dehydrocholesterol to previtamin D3, which is then isomerized to vitamin D3 (cholecalciferol), takes place [1]. Cholecalciferol, bound to vitamin D-binding protein in the blood, is subsequently hydroxylated in the liver to 25-hydroxyvitamin D (25(OH)D), which is then converted to the active hormone 1,25-dihydroxyvitamin D (1,25(OH)D, or calcitriol) by 1a-hydroxylase in the kidney [1].

Vitamin D is a recognized key factor in bone health and mineralization, where it acts as the main regulator of calcium and phosphate absorption [2]. Moreover, several studies have demonstrated the key role played by vitamin D in many other extraskeletal districts (i.e., cardiometabolic and neurodegenerative pathophysiology, and cancer) [1,3,4]. Although the measurement of blood levels of 25-hydroxyvitamin D (25(OH)D) is widely accepted as the best biomarker of vitamin D status [5], a definitive general consensus on how to define vitamin D deficiency has not yet been reached, especially with regard to extraskeletal conditions, such as cardiometabolic disease [1]. However, the recommendations of the United States Endocrine Society guidelines are usually followed, according to which vitamin D levels equal to or higher than 30 ng/mL (>75 nmol/L) are considered adequate [5]. Accordingly, hypovitaminosis D is generally defined for lower 25(OH)D levels; specifically, it is classified as vitamin D “insufficiency” when 25(OH)D levels range between 21 and 29 ng/mL (corresponding to 52.5–72.5 nmol/L), while “deficiency” occurs when levels are less than 20 ng/mL (<50 nmol/L). The deficiency then becomes a “severe deficiency” for 25(OH)D values below 10 ng/mL (<25 nmol/L) [5].

The pathophysiological mechanisms through which vitamin D can affect the cardiovascular (CV) system are indeed numerous. Among these processes, some are related to endothelial function (where an increase in nitric oxide availability and improvement in vasodilation and blood pressure are considered the main effects), others to inflammatory responses (e.g., a reduction in pro-inflammatory cytokines and an increase in anti-inflammatory ones), and others to improvement in mitochondrial function and insulin resistance [1]. In addition, effects on cardiac fibrosis, cardiomyocyte proliferation, and immune responses have also been reported [1].

Vitamin D has also been found to be inversely correlated with several biomarkers of oxidative stress and inflammation (e.g., different interleukins, oxidized low-density lipoproteins, malondialdehyde, and homocysteine), along with findings of antioxidant depletion (e.g., total antioxidant capacity, paraoxonase-1, and arylesterase) with hypovitaminosis D in subjects with cardiovascular risk factors (e.g., obesity, and type 2 diabetes) or coronary artery disease [1]. In patients with acute myocardial infarction (AMI), an acute event characterized by myocardial ischemia leading to myocardial injury, vitamin D levels are inversely correlated to biomarkers of plaque vulnerability, such as metalloproteinase-2 expression and leptin blood levels [6].

In particular, while subjects with serum hypovitaminosis D (25-hydroxyvitamin D3, 25(OH)D < 20 ng/mL) have a higher CV risk, patients with AMI are characterized by a severe deficiency in 25(OH)D levels, which also correlates with post-infarction complications, increased mortality, and adverse cardiovascular events in this clinical setting [1,7,8]. Specifically, in a previous work, we observed that more than 80% of AMI patients had suboptimal 25(OH)D levels, a finding that was confirmed in many other cohorts [9,10]. Indeed, a meta-analysis, which included 6123 CV disease (CVD) cases from 65994 participants, reported an adjusted pooled relative risk of 1.52 (95% confidence interval, 1.30–1.77) for total CV events when comparing the lowest with the highest 25(OH) concentration [10].

Furthermore, a recent meta-analysis (79 studies; 46 713 CVD cases in 1 397 831 participants) showed a linear correlation between every 10 ng/mL increase in 25(OH)D and non-fatal CVD incidence events (RR = 0.94; 95% CI = 0.89–0.98, *p* ≤ 0.01), lower fatal recurrent CVD events (RR = 0.45; 95% CI = 0.32–0.62, *p* < 0.001), and lower combined recurrent CVD events (RR = 0.80; 95% CI = 0.65–0.97, *p* < 0.05) [11].

Sphingolipids (SLs) are integral components of membranes, and their structure is characterized by a hydrophilic region (with phosphate groups, sugar residues, and/or hydroxyl groups) and a hydrophobic region (with sphingoid or long-chain bases) [12]. The main functionally active molecules among sphingolipids are sphingomyelin (consisting of a phosphocholine head group, a sphingosine, and a fatty acid), ceramides (characterized by a sphingoid base linked by an amide bond to a fatty acid), sphingosine (an 18-carbon amino alcohol with an unsaturated hydrocarbon chain, which constitute the sphingolipid backbone), and sphingosine-1-phosphate (S1P; sphingolipid with a phospho-group) [13]. Ceramides, which are the structural building blocks for the production of other more complex sphingolipids, are primarily generated through three main pathways that individually or mutually contribute to the production of these molecules: the de novo pathway, the sphingomyelin pathway, and the salvage/recycling pathway [12]. In brief, the pathways are as follows:

(a) In the de novo pathway, which takes place in the cytosolic layer of the endoplasmic reticulum, the enzyme serine palmitoyl-CoA transferase leads to the production of 3-keto-dihydrosphingosine through the condensation of palmitoyl-CoA and serine. Then, sphinganine is synthesized from 3-keto-dihydrosphingosine via the 3-keto-dihydrosphingosine reductase, while dihydroceramides are produced from different isoforms of ceramide synthase through the addition of acyl-CoA with different chain lengths into the sphinganine molecule. Once synthesized, ceramides are released by a dihydroceramide desaturase, transported into the Golgi, and then metabolized into complex sphingolipids (e.g., sphingomyelin, glucosylceramides, and gangliosides) or phosphorylated by the ceramide kinase to generate ceramide-1-phosphate [13]. 

(b) In the sphingomyelinase pathway: ceramide and phosphocholine are produced through the hydrolysis of sphingomyelin catalyzed by neutral or acidic sphingomyelinases. 

(c) The salvage pathway takes place in endo-lysosomes, where ceramides are generated from sphingosine through ceramide synthase, while the ceramidase enzyme leads to the production of sphingosine and a fatty acid through the hydrolysis of ceramide. Then, sphingosine kinase produces S1P, whereas sphingosine phosphatase can generate sphingosine [13].

These molecules exhibit strong biological properties in several key cellular pathways (e.g., apoptosis, cell proliferation, inflammatory responses, the activities of enzymes); the number of effects elicited depends on their chemical structure (which can greatly vary according to the length of the fatty acyl chain) and on the cellular compartment location [12]. Moreover, SLs have gained scientific interest due to their emerging role in the pathophysiology of cardiometabolic diseases (e.g., type 2 diabetes, acute and chronic coronary artery disease, hypertension, and stroke) [14]. In particular, they play important roles in endothelial dysfunction, mainly by reducing nitric oxide bioavailability; other data suggest that ceramides may induce vasoconstriction by increasing intracellular calcium concentration in isolated canine cerebral arterial smooth muscle [14]. Other experimental studies and clinical trials corroborate the relationship between sphingolipids and hypertension [14]. Furthermore, ceramides have been associated with increased mitochondrial damage and dysfunction, inflammation, oxidative stress, insulin resistance, and type 2 diabetes [15].

In atherosclerotic-related processes, SLs play a direct role in plaque formation and progression (e.g., by inducing low-density lipoprotein oxidation and affecting the phenotype of vascular smooth muscle cells) [16]. In a previous work, we highlighted how specific ceramide species are linked to cardiovascular risk, inflammation, and disease extent in AMI [17]. Other data also evidenced that ceramides and phospholipid-based risk scores are independently associated with adverse prognosis (cardiovascular death) in patients after acute coronary syndrome [18]. 

Interestingly, the measurement of ceramides, in addition to high-sensitivity cardiac troponins and traditional risk factors, showed great potential in the identification of acute coronary syndrome among patients with chest pain. In particular, after adjusting for traditional risk variables and high-sensitivity cardiac troponin T, Cer(d18:1/14:0), Cer(d18:1/22:0), and their ratio were found to be independent predictors of ACS. 

When comparing the multivariable model with ceramides to that without, the receiver operating characteristic curve analysis revealed a substantial improvement in ACS detection (area under the curve (AUC) of 0.865 (0.840 to 0.889) vs. 0.808 (0.776 to 0.841), *p* < 0.001) [19].

Additionally, the levels of sphingolipids (including ceramides and sphingomyelin) measured in homogenates from 200 human carotid plaques were associated with inflammation and plaque instability (association with symptoms, levels of inflammatory cytokines, and histological biomarkers of plaque instability) [20]. 

Another experimental study indicated that the overexpression of acid ceramidase (an enzyme that hydrolyzes ceramide into sphingosine and fatty acid) may modulate sphingolipid metabolism, reducing cell death and the inflammatory response after AMI [21].

In addition to their shared targets (e.g., inflammatory and oxidative responses, cell proliferation and growth, insulin resistance, obesity, and type 2 diabetes), a growing body of literature indicates the existence of mutual crosstalk between vitamin D and sphingolipids. Specifically, vitamin D can affect sphingolipid metabolism at different levels (e.g., by modulating the hydrolysis of sphingomyelin; modulating S1P receptors and sphingosine kinase 1 and 2 expression; downregulating ceramide kinase, which is responsible for the synthesis, expression, and content of ceramide-1-phosphate; and upregulating ceramide). Experimental and clinical data, indeed, show how vitamin D administration can modify the sphingolipid profile in type 2 diabetes, dyslipidemia, and overweight/obese subjects [22]. Some studies have also indicated a role for sphingolipid localization and expression in lipid rafts (lipid-rich microdomains) that may affect vitamin D receptor localization and functioning [22].

Recently, research in the cardiometabolic field as well as on the nervous system has explored this reciprocal relationship between vitamin D and SLs. These studies reported significant implications both for basic science, which aims to explain the mechanisms that elicit protection, and for the development of novel therapeutic approaches that involve vitamin D supplementation and other molecules that can modulate sphingolipids, such as agonists or antagonists of the S1P receptor [22,23].

However, since no information is available regarding the link between serum 25(OH)D and ceramides in AMI, we aimed to assess their relationship in this specific clinical setting.

## 2. Materials and Methods

### 2.1. Studied Population

A total of 134 patients (aged 68.4 ± 12.0 years, 97 males) with acute ST-elevation myocardial infarction (STEMI, an AMI event characterized by transmural myocardial ischemia resulting in myocardial injury or tissue necrosis) were admitted to the Cardiology Unit of the Ospedale del Cuore G. Pasquinucci (Massa, Italy) and their data were obtained from the Hospital’s computerized database [10]. STEMI was defined according to the SC/ACCF/AHA/WHF guidelines for STEMI criteria and management [24]. Within 90 min after admission to the intensive care unit, patients underwent coronary angiography, followed by percutaneous coronary intervention. CV risk factors were defined as follows: overweight or obesity, for body mass index higher than 25 kg/m^2^; hypertension, in case of blood pressure higher than 140/90 mmHg and/or current use of antihypertensive drugs; dyslipidemia (DYSL) in presence of lipid-lowering treatments and/or fasting low-density lipoprotein levels higher than 150 mg/dL; type 2 diabetes (T2D), in case of antidiabetic treatment and/or fasting glucose higher than 126 mg/dL (7 mmol/L) on two separate tests before the acute event/finding of HbA1c higher than 6.49%; smoking history, determined through confirmation of current or former smoking habit by clinical anamnesis.

The inclusion criteria were as follows: (1) adult patients of both sexes; (2) subjects admitted to the Hospital Coronary Care Unit for chest pain, in which AMI was subsequently proven by coronary angiography; (3) informed consent signed by each patient (or by their relatives if the patient was unable to sign it). 

The exclusion criteria were as follows: (1) patients with active infection, inflammatory diseases (i.e., rheumatoid arthritis), and malignancies; (2) vitamin D supplementation; (3) a lack of a written informed consent.

Eligible patients received standard medical therapy, which included the administration of aspirin, beta-blockers, ACE-inhibitors, diuretics, and anti-diabetic and lipid-lowering drugs. 

### 2.2. HPLC-MS/MS Analysis for Ceramide Assessment

#### 2.2.1. Plasma Processing for Ceramide Assessment

**Figure 1:**
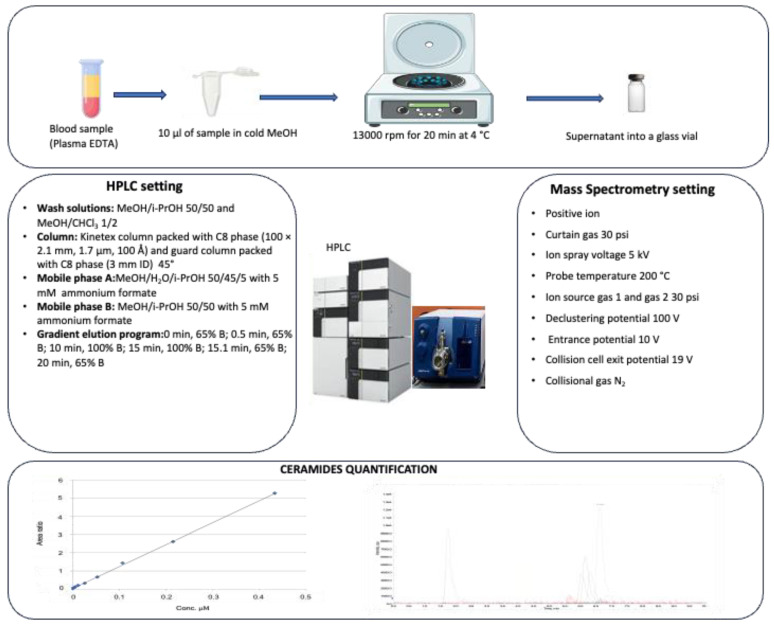
Schematic representation of sample preparation, HPLC, and mass spectrometry settings for ceramide quantification.

Plasma samples were stored at −80 °C, thawed at room temperature, and immediately subjected to lipid extraction and analysis: 200 μL of cold methanol, containing N,N-dimethylsphingosine (d18:1, DMS) 0.003 μM as the internal standard (ISTD), were added to 10 μL of plasma sample. After protein precipitation, the sample was centrifuged (13,000 rpm for 20 min at 4 °C) and the supernatant was analyzed by high-performance liquid chromatography–tandem mass spectrometry (HPLC-MS/MS) analysis (ISTD concentration of 0.00285 μM in the vial) (Figure 1) 

#### 2.2.2. Chemicals and Materials 

All chemicals, standards, and stock solutions used in this work have been previously reported [17]. In brief, ammonium formate eluent additive for LC-MS was purchased from Fluka Analytical (Sigma-Aldrich, St. Louis, MO, USA). LiChrosolv methanol and propan-2-ol, both hypergrade solvents for LC-MS, were purchased from Supelco (Merck KGaA, Darmstadt, Germany), while chloroform (for HPLC ≥ 99.8%) and DMSO were purchased from Sigma-Aldrich (St. Louis, MO, USA). Milli-Q deionized water was filtered on a Millipak filter (0.22 μm, MPGL040001) and purified on an LC-Pak cartridge (C18, LCP AK0001) (all Millipore, Bedford, MA 01730, USA). Ceramide calibration standard (Cer(d18:1/17:0)) and N,N-dimethylsphingosine (d18:1) were selected as internal standards (ISTD) and both purchased from Avanti Polar Lipids (Alabaster, AL, USA). The stock working solutions were prepared by dissolving Cer(d18:1/17:0) in CHCl3/MeOH 70/30, and N,N-dimethylsphingosine in DMSO (obtaining, as final concentrations, 1.5 mM and 15.3 mM, respectively).

#### 2.2.3. Cer(d18:1/17:0) Calibration Curve Preparation

ISTD stock solution was diluted with MeOH, to reach a final concentration of 0.057 μM. Nineteen calibration levels, ranging from 222.2 to 0.00085 μM, were prepared for Cer(d18:1/17:0), according to the following procedure: 29.6 μL of calibration STD stock solution was added to 160.4 μL of MeOH (final volume 190 μL). This solution was split into two aliquots of 95 μL each. An amount of 5 μL of 0.057 μM DMS was added to the first aliquot, thus creating the first calibration level (222.2 μM calibration STD, 0.00285 μM ISTD), while the second one was diluted two-fold with MeOH. The latter solution was, in turn, split into two aliquots of 95 μL each, and sequential two-fold dilutions were carried out till the 19th calibration level (0.00085 μM calibration STD, 0.00285 μM ISTD). Finally, a specific range of 0.00085–0.434 μM for the calibrators was selected in order to build an external calibration curve for Cer (d18:1/17:0) [25].

### 2.3. 25(OH)D Quantitative Measurements

Assessment of 25(OH)D was performed by DiaSorin “LIAISON 25-OH Vitamin D TOTAL CLIA”, a direct competitive immunochemiluminescent assay, as we previously described in detail on a fully automated LIASON Xl instrument for laboratory clinical activity. The method does not require sample pretreatment (it only requires a minimum sample of 250 μL, with a measuring interval of 4–150 ng/mL, a turn-around time of 40 min, and an assay throughput of 80 tests/h). The test is based on the following steps: (1) during the first incubation phase, 25(OH)D is separated from its binding protein, and it interacts with binding sites on the solid phase; (2) after the second incubation with the tracer, unbound material is washed off and a flash chemiluminescent signal is generated by adding the starter reagents, and finally, measured by a photomultiplier. The levels of 25(OH)D were defined, according to the Endocrine Society clinical practice guidelines, as follows: 25(OH)D sufficiency as a value equal or superior to 30 ng/mL, and vitamin D insufficiency/deficiency as 25(OH) D below 30 ng/mL, with severe deficiency for 25(OH)D values under 10 ng/mL [26]. The precision intervals (CV%) were as follows: within run (7–11%) and total precision (8–11.5%); mean (SD) recovery was 96 (2)% [26].

### 2.4. Statistical Analysis

Continuous variables are reported as mean ± SD, whereas categorical parameters are shown as numbers (percentage). Statistical analysis included Student’s t test (to assess whether the difference between two groups is statistically significant or not, comparing the means of two continuous variables), a χ2 test (for comparisons between categorical variables, to test whether the data observed and those expected correspond or not), and an analysis of variance (ANOVA: to evaluate whether there are any statistically significant differences between the means of different groups). Simple regression analysis was performed to verify the relationship between continuous variables and the degree of association measured by r (correlation coefficient), as a measure of linear association. Multivariable (multiple) regression analysis was also used to analyze the relationship between a single dependent variable and different independent variables, evidencing each relative contribution to the overall prediction; in particular, it was performed here to assess the effect of 25(OH)D and clinical and demographic variables in determining Cer(d18:1/16:0) and Cer(d18:1/18:0) levels.

A post hoc power analysis was performed using the program G*Power 3.1, choosing an effect size corresponding to 0.3. Considering this effect size, a study power (1-β) of 0.955 was expected, with an α value of 0.05.

The statistical package Statview, version 5.0.1 (SAS Institute, Abacus Concept, Inc., Berkeley, CA, USA), was used to perform the analyses. A *p* value, indicating the probability that any observed difference between groups is due to the case or not, of 0.05 or less was considered significant.

## 3. Results

The demographic and clinical characteristics of the AMI population are summarized in Table 1. According to the higher incidence of AMI in men than in women, we found that 97 (72%) of enrolled patients were males, and the mean ages among the two sexes were 66.3 ± 11.4 and 74 ± 11.9 years, respectively.

**Figure 2:**
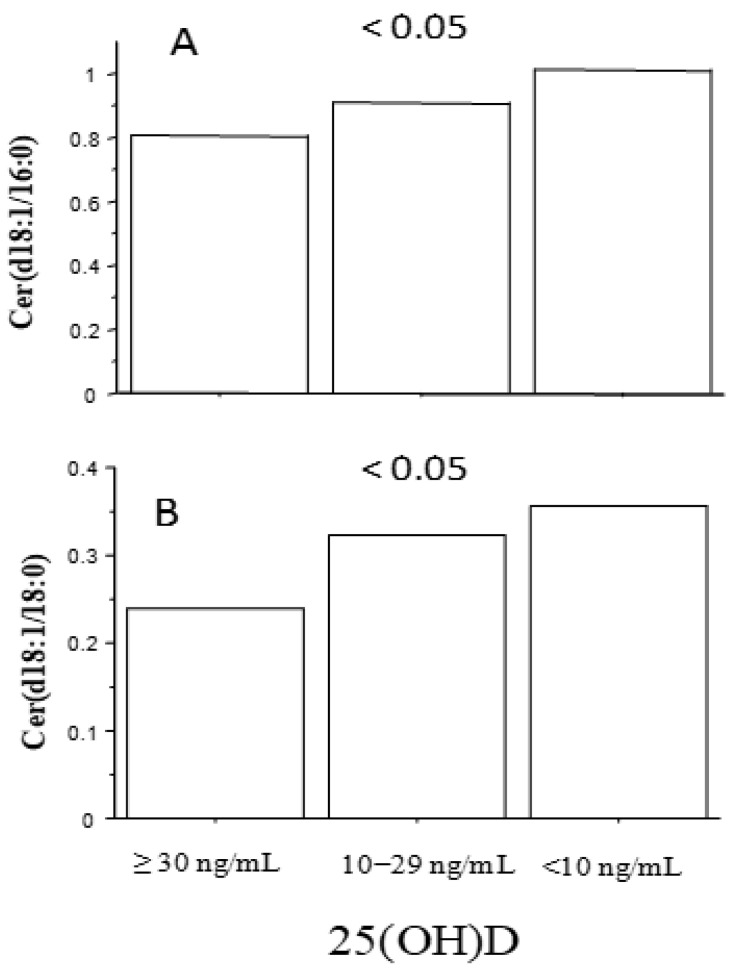
Levels of ceramides according to 25(OH)D levels (≥30, 10−29, <10 ng/mL); results for Cer(d18:1/16:0) and Cer(d18:1/18:0) in panel (**A**) and (**B**), respectively. (ANOVA analysis, *p* for trend).

**Figure 3:**
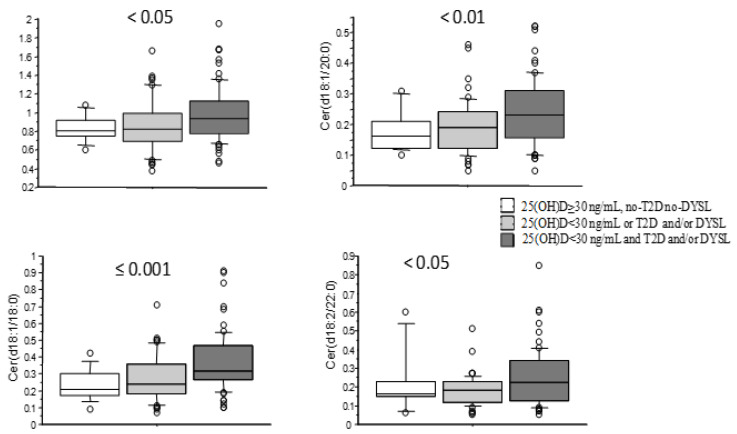
Levels of ceramides according to 25(OH)D levels and the presence of T2D and DYSL in patients with AMI. Those with adequate 25(OH)D levels and without T2D or DYSL have the lowest ceramide levels; patients with suboptimal 25(OH)D (without T2D or DYSL) or with T2D and/or DYSL (and adequate 25(OH)D) have intermediate levels; and patients with suboptimal 25(OH)D and T2D and/or DYSL have the highest ceramide levels (ANOVA analysis, *p* for trend).

Simple regression analysis evidenced a significant negative relationship between 25(OH)D and the two metabolites [Cer(d18:1/16:0): r = −0.18, *p* < 0.05 and Cer(d18:1/18:0): r = −0.2, *p* < 0.05] (Table 2). AMI patients with adequate 25(OH)D levels (≥30 ng/mL) showed reduced values of Cer(d18:1/16:0) and Cer(d18:1/18:0), which were particularly high in patients with severe hypovitaminosis D (<10 ng/mL) (0.8 ± 0.18, 0.9 ± 0.28, 1 ± 0.32 µmol/L, ANOVA: *p* < 0.05 and 0.24 ± 0.1, 0.32 ± 0.16, 0.36 ± 0.18, ANOVA: *p* < 0.05, respectively, for 25(OH)D ≥ 30, 10−29, <10 ng/mL) (Figure 2A,B). Moreover, the levels of the two metabolites were higher in patients with reduced 25(OH)D (<30 ng/mL), T2D, and/or DYSL (0.83 ± 0.14, 0.86 ± 0.27, 0.98 ± 0.29 µmol/L, ANOVA: *p* < 0.05; 0.24 ± 0.09, 0.27 ± 0.14, 0.36 ± 0.17 µmol/L, ANOVA: *p* ≤ 0.001; 0.17 ± 0.07, 0.19 ± 0.09, 0.24 ± 0.11 µmol/L, ANOVA: *p* < 0.01 f; 0.23 ± 0.16, 0.18 ± 0.08, 0.25 ± 0.15 µmol/L, ANOVA: *p* < 0.05 for the following categories: 25(OH)D ≥30 ng/mL no-T2D no-DYSL; 25(OH)D < 30 ng/mL (without T2D or DYSL ) or T2D and/or DYSL (and adequate 25(OH)D); 25(OH)D < 30 ng/mL and T2D and/or DYSL) (Figure 3). 

The multiple regression analysis for Cer(d18:1/16:0), controlled for 25(OH)D levels and all the demographic and clinical variables reported in Table 1, showed that 25(OH)D levels (STD Coeff −0.18, *t*-Value −2, *p* ≤ 0.05) and DYSL (STD Coeff 0.19, *t*-Value 2.2, *p* < 0.05) remained independent determinants affecting Cer(d18:1/16:0) in our population; for Cer(d18:1/18:0), 25(OH)D levels (STD Coeff −0.2, *t*-Value −2.2, *p* < 0.05), DYSL (STD Coeff 0.23, *t*-Value 2.6, *p* < 0.01), and male gender (STD Coeff −0.17, *t*-Value −2, *p* ≤ 0.05) remained independent factors after controlling for different cardiometabolic risk factors (Table 3 and Table 4).

## 4. Discussion

This study demonstrated, for the first time, that 25(OH)D affects the levels of particular ceramide species, especially for the higher-risk biomarkers previously reported, Cer(d18:1/16:0) and Cer(d18:1/18:0) [27].

In 2016, the first evidence was published indicating the presence of elevated ceramides (including Cer(d18:1/16:0) and Cer(d18:1/18:0)) in arterial plaque, inducing apoptosis/necrosis, as well as a sustained proinflammatory state, in human coronary artery smooth muscle cells [20]. One year later, ceramides were visualized in human coronary plaques, both ex vivo and in vivo, by fluorescent angioscopy [28]. Moreover, other studies also highlighted a relationship between some ceramides and major adverse cardiovascular events in acute and stable coronary artery disease patients. This correlation persists even after adjustment for traditional risk factors (in particular, over and above traditional lipid biomarkers, currently used in clinical practice), with Cer (d18:1/16:0), Cer(d18:1/18:0), Cer(d18:1/24:1), and Cer(d18:1/24:0) having the strongest relationship with major adverse cardiovascular events compared to all the other ceramides considered [29,30]. Further data also showed that ceramide concentration was higher in patients with plaque rupture compared to those with plaque erosion, in agreement with previous studies [28,31,32,33]. Taken together, these results highlight the importance of Cer in plaque phenotype, complexity, and vulnerability. Specifically, circulating levels of Cer(d18:1/16:0), Cer(d18:1/18:0), Cer(d18:1/24:1), and Cer(d18:1/24:0) were higher in STEMI patients compared to normal donors and stable patients; moreover, ceramides were significantly higher in STEMI patients with plaque rupture than in those with plaque erosion [31].

Cer(d18:1/16:0), Cer(d18:1/18:0), and Cer(d18:1/24:1) were also independently associated with the SYNTAX SCORE (which evaluates the complexity of coronary artery disease and is defined as the sum of the points assigned to each individual lesion identified in the coronary tree with >50% diameter narrowing in vessels >1.5 mm in diameter) in STEMI patients and, as such, with coronary atherosclerotic burden [32].

Other data suggested that ceramides, in particular, Cer (d18:1/16:0) and Cer(d18:1/24:0), may be associated with the characteristics of coronary plaque, such as the fraction of necrotic core tissue and lipid core burden (intravascular ultrasound virtual histology imaging, coronary lipid core burden index on near-infrared spectroscopy) [33]. Moreover, they are predictive of the 1-year clinical outcome in patients with coronary artery disease (all-cause mortality, acute coronary syndrome, and unplanned coronary revascularization) following coronary angiography [34]. In this context, while Cer(d18:1/16:0) has been linked to the progression of carotid artery plaques, immune activation crosstalk, and inflammation (monocyte activation, inflammation markers, and surface markers of CD4^+^ T-cell activation) in humans, sphingomyelin(34:1) (a Cer(d18:1/16:0) precursor) has been associated with increased carotid artery intima-media thickness in animal models [35,36].

Moreover, in Chinese patients with acute coronary syndrome, Cer(d18:1/16:0) and Cer(d18:1/18:0), as well as other forms of Cer, were linked to type 2 diabetes [37]. The analysis of FINRISK 2002 evidenced that blood concentrations of Cer(d18:1/16:0), Cer(d18:1/18:0), and Cer(d18:1/24:1) were associated with the development of insulin resistance and type 2 diabetes. In this context, it is worth noting the possibility of modulating this relationship (for example, via lifestyle interventions), which decreased in individuals who lost at least 5% of their body weight in this study [38,39]. Several other drugs may also act on ceramide levels; for example, pioglitazone (an antidiabetic drug) is able to significantly reduce the levels of high-risk ceramide, thus inducing beneficial modifications in insulin resistance and adiponectin levels.

In this context, statins can also reduce the levels of high-risk ceramides (including Cer(d18:1/16:0) and Cer(d18:1/18:0)), suggesting that different drugs used in the cardiometabolic field may modulate the sphingolipid profile [40,41,42]. Specifically, rosuvastatin (10 or 40 mg/d, respectively, for 5 weeks) significantly decreased circulating levels of sphingolipids and phospholipids (ceramide: −33 and −37%, respectively) in men with metabolic syndrome [40]. In the Ludwigshafen Risk and Cardiovascular Health (LURIC) study, healthy subjects treated with simvastatin (40 mg for two weeks) resulted in a 25% decrease in plasma ceramides [41]. Moreover, PCSK9 inhibition (RG7652, twenty-nine-day treatment in different dose regimens, all effective in lowering low-density lipoproteins) significantly decreased blood levels in different lipid classes, including sphingolipids such as dihydroceramide, glucosylceramides, sphingomyelins, ceramides (Cer(d18:1/16:0) and Cer(d18:1/18:0) levels decreased more than 30%), cholesteryl esters, and free cholesterol [42].

In addition to the numerous effects, of vitamin D on cardiometabolic conditions (e.g., oxidative stress and inflammation), its impact on the homeostasis of sphingolipids and ceramides at different levels of their metabolism is emerging as a potential additional key mechanism that affects cardiometabolic pathophysiology [1,22]. In vitro results suggested that vitamin D deficiency may affect the phospholipid composition of the rat renal brush border membrane and their fatty acid composition, while vitamin D administration (1,25(OH)D for 16 h) may partially restore these changes [43]. Furthermore, in an in vitro model, vitamin D was found to activate the sphingomyelin pathway, raising ceramide levels while decreasing those of sphingomyelin [44].

A recent interesting study also investigated the effect of vitamin D3 treatment on the exosomes released from embryonic hippocampal cells: here, an increase in neutral sphingomyelinase and changes in sphingomyelin were observed. Moreover, cell incubation with neutral sphingomyelinase, or ceramide (at the same concentration as that produced in exosomes), stimulated the differentiation of embryonic hippocampal cells in the same way as vitamin D3 [45]. This result highlights the link between the release of exosomes with a high ceramide content following vitamin D administration and the effect of vitamin D on cell differentiation, which may be, in turn, mediated by the sphingolipid composition of exosomes [45].

However, given the existence of several different groups of lipids, as well as intriguing mechanisms, the relationship between vitamin D and sphingolipids is far from completely understood. The action of vitamin D on sphingolipids may even be opposite in different districts, suggesting responses specific to particular cell types (e.g., the difference in the gene expression of sphingosine 1-phosphate receptor 3 (S1P3) between monocytes and human breast cancer cells) [46,47]. Moreover, it is known that the activity of the ceramide synthase enzyme isotype 2 (CerS2; C24:0 or 24:1) results in very long-chain ceramides, while other isoforms (CerS6, CerS5) produce long-chain crosstalk (e.g., C16:0). Nonetheless, it is also known that the haplo-insufficiency of CerS2 may drive a compensatory increase in C16:0, suggesting that, despite their direct production by different enzymes, ceramide homeostasis may be altered when ceramide pathways are changing; this may occur through compensatory mechanisms with the activation of possible negative side effects related to the production of more toxic sphingolipids [48,49].

It should also be considered that the link between vitamin D and sphingolipids may be bidirectional: indeed, changes in membrane sphingolipid composition might affect both the location and function of vitamin D receptors [50,51]. Moreover, several ceramide metabolites could enhance 1,25(OH)D-induced differentiation through the involvement of the PI3-K/PKC/JNK/ERK pathways, as seen in HL-60 human leukemia cellular models [52].

It is crucial to remember that any contentious results that exist between studies could be the result of additional methodological errors. Indeed, some investigations are performed in humans, while others are performed in animal models; also, while some studies focus on ceramide differences between acute patients (e.g., AMI) and subjects with stable coronary artery disease, others assess the difference between AMI patients and healthy individuals [53,54].

However, although data are still very limited, increasing findings have already established a link between hypovitaminosis D (and/or its supplementation) and changes in the sphingolipid profile in T2D, dyslipidemic, and obese subjects. These alterations may also involve several other metabolites (e.g., ceramides, phosphatidylcholines, lysophospholipids, phosphatidylethanolamines, and sphingomyelin) [22].

## 5. Limitations of this Study

Whether the 25(OH)D test is widely used in laboratory routines for clinical purposes, the evaluation of ceramides is not so widespread, because it is still expensive and requires specialized instruments, careful maintenance, and skilled operators with technical expertise [55]. However, ceramide testing implementation in clinical practice is possible, even in the very near future. A crucial aspect, however, is the comparability of results between assays and laboratories, as different methods for lipidomic analyses are used. In this context, developing tests for clinical usage requires close collaboration between manufacturers and users to create identical internal standards from the same source while refining reference values.

In our work, a control group that could be used to measure ceramides was not available. Hence, further information is required to determine the degree to which ceramide metabolism in AMI patients differs from that in the general population. Nonetheless, Cer (d18:1/16:0) and Cer(d18:1/18:0), which are the most studied ceramides in acute coronary syndrome studies, were generally higher in patients with AMI than in patients with other levels of coronary atherosclerosis and in comparison to healthy subjects [13,17,31,32]. Recently, marked changes in the lipidomic composition of HDL particle subpopulations in STEMI have been reported, particularly for Cer(d18:1/16:0) and Cer(d18:1/18:0) species, which were significantly increased in patients compared with healthy control subjects [56]. 

Although 25(OH)D did not change during the first 72 h after AMI hospitalization, the possibility of temporal variations in ceramide levels in this clinical setting requires further investigation in future studies.

The studied population was limited to Italian subjects, and thus, our results need further validation before being extended to other ethnic groups.

## 6. Conclusions

This work demonstrates, for the first time, the existence of mutual crosstalk between sphingolipid signaling and vitamin D in AMI, an interaction that may identify novel mechanisms by which these molecules can influence the CV risk in AMI patients.

Traditional lipids have represented, and always will represent, a cornerstone in the field of cardiometabolic disease; however, a deeper understanding of the metabolism, profile, and levels of sphingolipids may improve our comprehension of atherosclerosis pathophysiology, thus providing additional opportunities to develop new and more targeted therapeutic tools. Indeed, the sphingolipid pathway might be one of the targets through which positive effects observed with the supplementation of cholecalciferol and its analogs are explained.

Nevertheless, it is still unclear how this relationship contributes in a clinical context, and more studies are required to determine if the balance is advantageous or unfavorable for the development and prognosis of AMI. In fact, many yet unexplored aspects warrant additional investigations in the future to better understand the fundamentals of this relationship. In this context, further studies are required to understand the roles played by different types of sphingolipids (such as the biological effects of ceramides according to chain length). Additional investigations are also needed to elucidate the underlying mechanisms by which these molecules act toward inflammation and oxidative stress, identify additional key specific species that can act as effective biomarkers, and establish a better understanding on how their role in different districts may even be opposite (beneficial or adverse). Moreover, in addition to 1,25(OH)D, the known biological form of vitamin D, further attention needs to be paid to the biological effects and therapeutic potential of other metabolites of the vitamin D pathway.

Nevertheless, the possibility to better stratify patients who may benefit from specific interventions, for example, by combining the use of vitamin D supplementation with sphingolipid-targeted drugs and traditional drugs, may also provide a new tool with clinical relevance in the AMI field to develop more personalized AMI management plans that better meet the individual specific needs of each patient. These novel therapeutic strategies should also be tested for their efficacy in reducing complications and unfavorable prognoses in the post-AMI follow-up.

## Figures and Tables

**Table 1:** Characteristics of the studied AMI population.

Number	134
Age (>50th percentile, 69 y)	59 (44)
Females	37 (28)
T2D or dyslipidemia	77 (58)
Hypertension	82 (62)
Smoking history (current/ex-smokers)	60 (45)
Overweight/obesity (BMI > 25 kg/m^2^)	64 (48)

Data reported as n (%). AMI = acute myocardial infarction; T2D = type 2 diabetes; BMI = body mass index.

**Table 2:** Simple regression analysis between 25(OH)D and ceramide levels.

	25(OH)D	
	r (Correlation Coefficient)	*p* Value (Significance)
Cer(d18:1/16:0)	0.18	<0.05
Cer(d18:1/18:0)	0.2	<0.05
Cer(d18:1/20:0)	0.13	ns
Cer(d18:1/22:0)	0.11	ns
Cer(d18:1/23:0)	0.05	ns
Cer(d18:1/24:0)	0.01	ns
Cer(d18:1/24:1)	0.01	ns
Cer(d18:1/25:0)	0.01	ns
Cer(d18:2/22:0)	0.05	ns

**Table 3:** Multiple regression between Cer(d18:1/16:0) controlled for different cardiometabolic risk factors.

Variable	STD Coeff	*t*-Value	*p*-Value (Significance)
Age (yrs)	0.19	1.8	ns
Male gender	−0.09	−1	ns
T2D	−0.08	−0.9	ns
DYSL	0.19	2.2	<0.05
Hypertension	0.08	0.9	ns
Smoking history	0.1	1	ns
Overweight/obesity	−0.12	−1.4	ns
25(OH9D	−0.18	−2	≤0.05

T2D = type 2 diabetes; DYSL = dyslipidemia.

**Table 4:** Multiple regression between Cer(d18:1/18:0) controlled for different cardiometabolic risk factors.

Variable	STD Coeff	*t*-Value	*p*-Value (Significance)
Age (yrs)	−0.01	−0.13	ns
Male gender	−0.17	−2	≤0.05
T2D	0.03	−0.3	ns
DYSL	0.23	2.6	<0.01
Hypertension	0.1	1.1	ns
Smoking history	0.04	0.4	ns
Overweight/obesity	−0.07	−0.8	ns
25(OH9D	−0.2	−2.2	<0.05

T2D = type 2 diabetes; DYSL = dyslipidemia.

## Data Availability

Data are available on request to authors. We have no permission from the ethical committee to share the data of patients. The data are in an institutional dataset and available on request from the clinicians who take care of the data.
